# Chromatin remodeler ARID1A binds IRF3 to selectively induce antiviral interferon production in macrophages

**DOI:** 10.1038/s41419-021-04032-9

**Published:** 2021-07-27

**Authors:** Ye Hu, Xin Wang, Jiaying Song, Jiacheng Wu, Jia Xu, Yangyang Chai, Yuanyuan Ding, Bingjing Wang, Chunmei Wang, Yong Zhao, Zhongyang Shen, Xiaoqing Xu, Xuetao Cao

**Affiliations:** 1 Department of Immunology, Institute of Basic Medical Sciences, Peking Union Medical College, Chinese Academy of Medical Sciences, Beijing, China; 2 CAMS-Oxford Translational Institute, Chinese Academy of Medical Sciences, Beijing, China; 3 Suzhou Institute of Systems Medicine, Suzhou, China; 4 Fuwai Central China Cardiovascular Hospital, Heart Center of Henan Provincial People’s Hospital, Zhengzhou, China; 5 Institute of Transplanation Medicine, First Central Hospital, Nankai University, Tianjin, China; 6 College of Life Sciences, Nankai University, Tianjin, China

**Keywords:** Interferons, Infection

## Abstract

Transcription factor IRF3 is critical for the induction of antiviral type I interferon (IFN-I). The epigenetic regulation of IFN-I production in antiviral innate immunity needs to be further identified. Here, we reported that epigenetic remodeler ARID1A, a critical component of the mSWI/SNF complex, could bind IRF3 and then was recruited to the *Ifn-I* promoter by IRF3, thus selectively promoting IFN-I but not TNF-α, IL-6 production in macrophages upon viral infection. Myeloid cell-specific deficiency of *Arid1a* rendered mice more susceptible to viral infection, accompanied with less IFN-I production. Mechanistically, ARID1A facilitates chromatin accessibility of IRF3 at the *Ifn-I* promoters by interacting with histone methyltransferase NSD2, which methylates H3K4 and H3K36 of the promoter regions. Our findings demonstrated the new roles of ARID1A and NSD2 in innate immunity, providing insight into the crosstalks of chromatin remodeling, histone modification, and transcription factors in the epigenetic regulation of antiviral innate immunity.

## Introduction

Chromatin remodeling plays a critical role in regulating gene transcription [1, 2]. Epigenetic factors remodel chromatin architecture to facilitate or inhibit the access of the transcription factors or regulatory proteins to the promoter region of genes, including those involved in the innate immune response and inflammation [3, 4]. As the most studied epigenetic regulators, histone modifications such as methylation, acetylation, and ubiquitination, are precisely regulated to control the transcription of immune-associated genes [5]. Among those most important cytokines, type I interferons (IFN-I) including IFN-α and IFN-β have strong antiviral activities and also play important roles in the pathogenesis of inflammatory diseases [4, 6, 7]. The methylation status (mono-, di- or tri-methylation) of specific lysine (K) residues in the histone N terminal are associated with gene activation or repression. For example, methylation of K4 or K36 of histone 3 (H3K4, or H3K36) promotes gene transcription, whereas methylation of H3K9 or H3K27 inhibits gene expression [8, 9]. Although di-/tri-methylation of K27 of histone (H3K27me2/3) was recently reported to promote IFN-β production in macrophages [10], the potential roles of histone modification in IFN-I signaling regulation are worth further investigation. Also, the crosstalk among multiple regulatory proteins and their synergistic effects in regulating transcription of *Ifn-I* during innate immune response remains to be fully understood.

BAF complex (Brg/Brahma-associated factors), known in mammals as the mSWI/SNF (SWItch Sucrose non-fermentable) complex, is an evolutionarily conserved ATP-dependent chromatin remodeler [11]. The mSWI/SNF complex has been intensively investigated in the context of stem cell development, the pathogenesis of the metabolic disease, and cancer. The mechanism for the action of the SWI/SNF complex was previously thought to regulate nucleosomes and facilitate chromatin accessibility after recruitment by transcription factors to the DNA promoter locus [12]. The mSWI/SNF complex has been reported to be essential for T cell development [13, 14]. Besides, a previous study showed that ATPase BRG1, the core component of the mSWI/SNF, could bind with NF-κB and activate *Ifnb* transcription upon viral infection [15]. As another key component of the mSWI/SNF complex, ARID1A (AT-rich interacting domain-containing protein 1 A, also known as BAF250a) binds DNA in a non-sequence-specific manner by its ARID domain to guide the location of the mSWI/SNF complex and is also involved in the protein–protein interactions via its C-terminal region [16]. However, the role of the ARID1A subunit in innate immunity remains poorly understood and is worth further investigation.

In our study, we investigated the role of ARID1A in the antiviral innate response and found that ARID1A can selectively promote IFN-I production in macrophages. We revealed that, in response to viral infection, ARID1A can be recruited by IRF3 at the *Ifn-I* promoters and interact with the protein methyltransferase NSD2 which methylates H3K4 (H3K4Me3) and H3K36 (H3K36Me2) to facilitate chromatin accessibility at the *Ifn-I* promoters, resulting in the increased transcription of *Ifn-I*. Therefore, we demonstrated the interplay among the nuclear proteins including chromotin remodeler ARID1A, transcriptional factor IRF3, and histone modification protein NSD2 at the *Ifn-I* promoter regions which jointly regulate the production of IFN-I in innate immune cells upon virus infection. Our findings about the promotion of *Ifn-I* transcription by ARID1A during innate response provide new insight to the epigenetic regulation of innate immunity.

## Results

### ARID1A selectively promotes IFN-I production in antiviral immune response

*Arid1a* expression was quite abundant in immune-related cells and tissues according to the BioGPS website (BioGPS.org). The higher expression level of *Arid1a* was verified in the spleen and lymph nodes of mice compared to other organs (Fig. S1A). Immunofluorescence assay and Western blot confirmed that ARID1A protein was located in the nucleus of macrophages (Fig. S1B, C). However, we found no significant change of ARID1A protein expression in peritoneal macrophages (PMs), bone marrow-derived macrophages (BMDMs), and RAW264.7 cells before and after infection with RNA virus such as vesicular stomatitis virus (VSV) and Sendai virus (SeV) or DNA virus herpes simplex virus 1 (HSV-1) (Fig. S1D–F). Similarly, ARID1A protein expression remained constant in splenocytes of mice after in vivo infection with VSV (Fig. S1G).

**Fig. 1:**
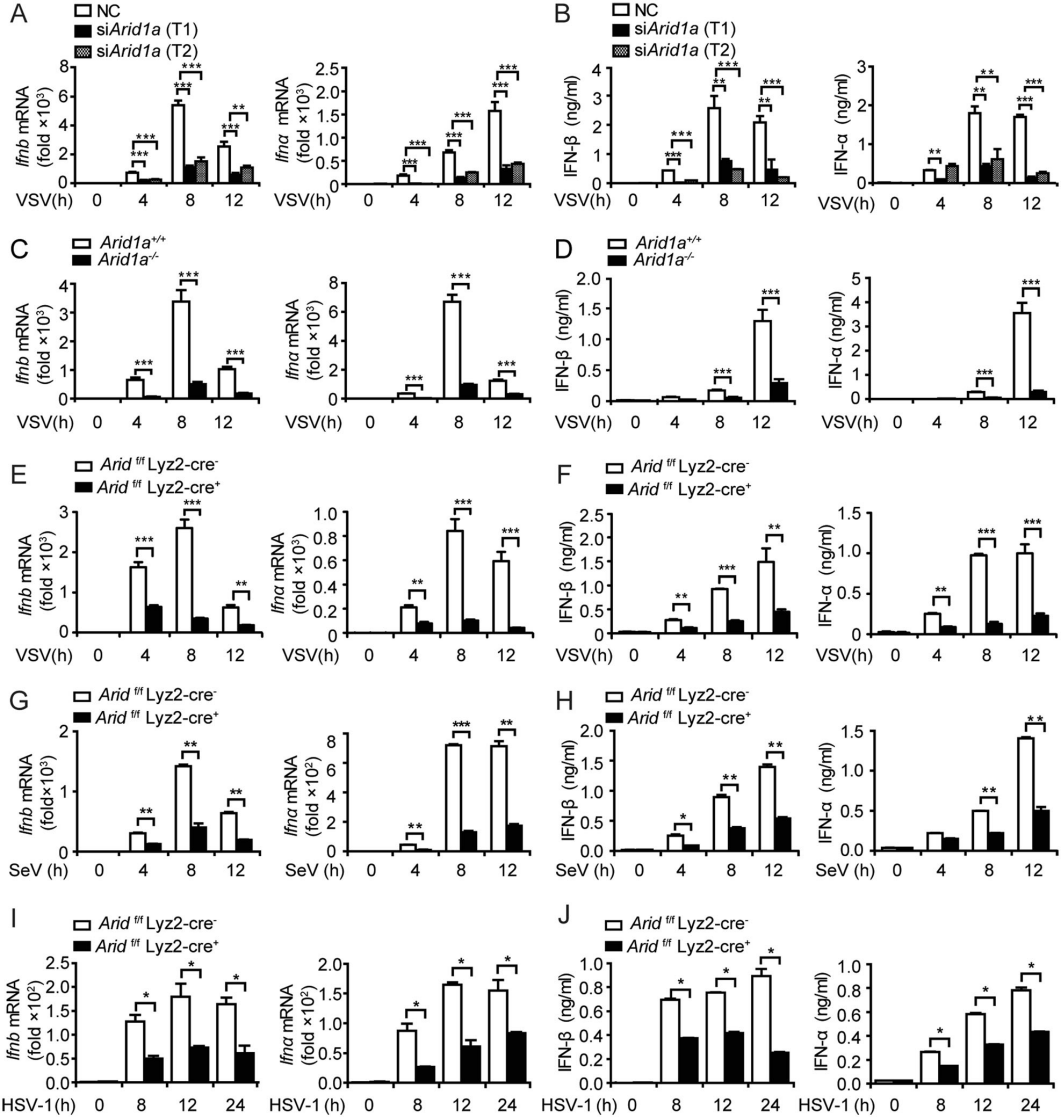
Inhibition of *Arid1a* suppresses IFN-I production upon virus infection. **A**–**D** qPCR analysis of *Ifn-I* (**A**, **C**) and ELISA analysis of IFN-I (**B**, **D**) expression in PMs silenced (**A**, **B**) by two specific *Arid1a* siRNAs or non-coding (NC) siRNA and *Arid1a* knock-out RAW264.7 (**C**, **D**) and control cells in response to VSV (MOI, 1) infection at the indicated times. **E**–**J** qPCR analysis of *Ifn-I* (**E**, **G**, **I**) and ELISA analysis of IFN-I (**F**, **H**, **J**) expression in *Arid1a* ^*f/f*^ Lyz-Cre^*-*^ and *Arid1a* ^*f/f*^ Lyz-Cre^*+*^ BMDMs in response to VSV (MOI, 1) (**E**, **F**), SeV (MOI, 1) (**G**, **H**) or HSV-1 (MOI, 10) (**I**, **J**) infection at the indicated times. Error bars represent s.d. Student’s *t*-test. *P < 0.05, **P < 0.01, ***P < 0.001. All data are representative of three independent experiments with three biological replicates. (**A**–**J**: mean ± s.d.).

Despite of the unchanged expression during the acute virus infection, we are curious about the function of ARID1A in antiviral immune response, considering its high abundance in immune-related cells. Therefore, we firstly interfered with *Arid1a* expression in PMs and examined the production of cytokines including IFN-α, IFN-β, TNF-α, and IL-6 in response to VSV infection. The expression of IFN-I (IFN-α and IFN-β) was significantly decreased in *Arid1a-*silenced PMs after VSV infection (Fig. 1A, B; Fig. S2A), whereas the expression of TNF-α and IL-6 remained unchanged (Fig. S2B, C). Then we generated *Arid1a* knock-out RAW264.7 cells using the CRISPR-Cas9 system (Fig. S2D). Compared with control cells, *Arid1a*-deficient RAW264.7 cells showed a substantial reduction of IFN-I but not TNF-α or IL-6 after VSV infection (Fig. 1C, D; Fig. S2E, F). Accordingly, the expression of interferon-stimulated genes (ISGs) such as *Ifit1*, *Cxcl10*, *Ccl5,* and *Mx1* also reduced in the *Arid1a* knock-out RAW264.7 cells infected with VSV (Fig. S2G). On the contrary, *Arid1a*-overexpressed RAW264.7 cells exhibited substantially increased IFN-I production in response to VSV infection (Fig. S2H, I and J). These data indicated that ARID1A selectively promotes IFN-I production in macrophages upon viral infection.

Next, we established myeloid cell-specific *Arid1a* conditional knock-out mice by crossing *Arid1a* flox/flox mice [17] with Lyz2-cre mice and confirmed the efficiency of *Arid1a* deletion in BMDMs (Fig. S3A, B). Previous studies have reported that deficiency of *Arid1a* does not affect the formation and stability of other subunits in the mSWI/SNF complex, but may influence the assembly and the recruitment of complex [18, 19]. Therefore, we examined the expression of other important subunits in the mSWI/SNF complex and the interaction between different subunits in both *Arid1a* -deficient (*Arid1a*^*f/f*^ Lyz-Cre^+^) mice and control (*Arid1a*^*f/f*^ Lyz-Cre^−^) mice. Indeed, no difference was observed between the two groups with or without VSV infection (Fig. S3C), which suggested Arid1a deletion in BMDMs has no impact on the integrity or stability of the mSWI/SNF complex.

Consistently, we discovered the expression of IFN-I and ISGs were substantially decreased in *Arid1a*-deficient BMDMs infected with VSV, SeV, or HSV-1 respectively (Fig. 1E–J; Fig. S3D). However, the expression of TNF-α and IL-6 in *Arid1a*-deficient BMDMs was comparable with that in control BMDMs upon virus infection (Fig. S3E–J). By RNA-seq analysis on the *Arid1a*-deficient BMDMs infected with VSV, we further confirmed the selective decrease of *Ifn-I* expression accompanied with *Arid1a* deletion. Meanwhile, the differentially expressed genes included multiple genes involved in virus-associated signaling pathways (Fig. S4A–D). Taken together, our data demonstrated that ARID1A selectively promotes IFN-I production in antiviral innate response.

### ARID1A protects mice from virus infection by promoting IFN-I production

FACS analysis showed that the ratios of splenic CD4^+^ T cells, CD8^+^ T cells, B cells, macrophages, dendritic cells, NK cells, neutrophils, and eosinophils were all comparable between *Arid1a* ^*f/f*^ Lyz-Cre^*+*^ mice and littermate controls (Fig. S5A and B), indicating deletion of *Arid1a* in myeloid cells has no effect on the development of immune cells. We also confirmed that the generation of BMDMs and PMs were not affected in *Arid1a*-deficient mice (Fig. S5C).

**Fig. 2:**
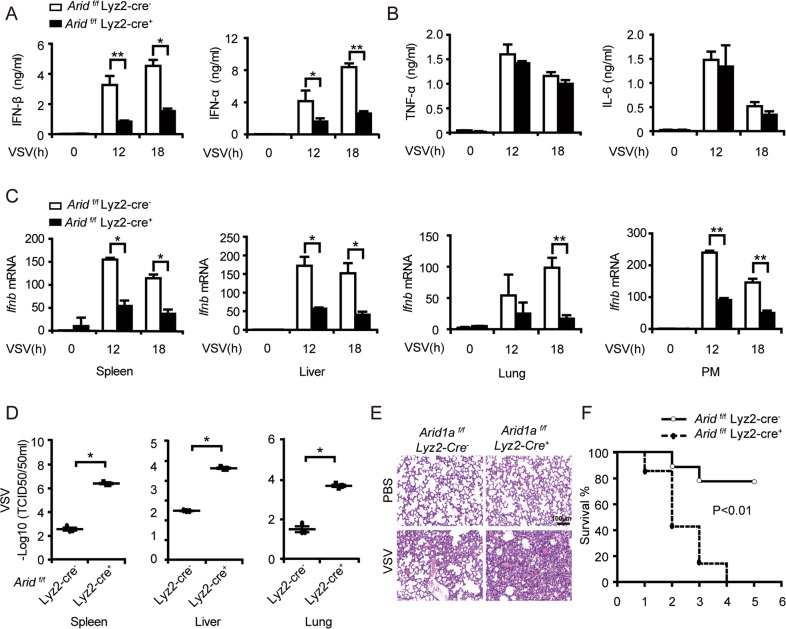
*Arid1a*-deficient mice are more susceptible to VSV infection. **A** ELISA analysis of IFN-I in the serum of *Arid1a* ^*f/f*^ Lyz-Cre^*−*^ and *Arid1a* ^*f/f*^ Lyz-Cre^*+*^ mice infected with VSV (5 × 10^7^ pfu/g, *n* = 3 per group) at indicated times. **B** ELISA analysis of TNF-α and IL-6 in the serum of mice treated as in (**A**). (**C**) qPCR analysis of *Ifnb* expression in spleen, lung, liver and PMs of mice treated as in (**A**). **D** TCID50 assay of VSV load in spleen, lung, and liver of *Arid1a* ^*f/f*^ Lyz-Cre^*−*^ and *Arid1a* ^*f/f*^ Lyz-Cre^*+*^ mice 18 h after VSV infection (5 × 10^7^ pfu/g, *n* = 3 per group). **E** HE staining of the lung tissue from mice treated as (**D**) (scale bar: 100 μm). **F** Survival curve analysis of *Arid1a* ^*f/f*^ Lyz-Cre^*−*^ and *Arid1a* ^*f/f*^ Lyz-Cre^*+*^ mice intravenously injected with VSV (1 × 10^8^ pfu/g, *n* = 7 per group). Kaplan–Meier survival curves were generated and analyzed for statistical significance with GraphPad Prism 5.0. Error bars represent s.d. Student’s *t*-test. *P < 0.05, **P < 0.01. All data are representative of three independent experiments with three biological replicates. (**A**–**D**: mean ± s.d.).

After challenging *Arid1a*-deficient mice and control mice by intravenously injecting VSV, we found that the production of IFN-I in the sera of *Arid1a*-deficient mice was much lower than those in control mice (Fig. 2A), whereas the production of TNF-α and IL-6 were comparable (Fig. 2B). We also detected the significantly decreased *Ifnb* mRNA expression as well as increased VSV titer in the spleen, liver, lung, and PMs of *Arid1a*-deficient mice compared to their littermate controls (Fig. 2C, D). Consequently, the obviously increased infiltration of inflammatory cells into the lung of *Arid1a*-deficient mice was observed (Fig. 2E). Moreover, the lethality of VSV infection in *Arid1a*-deficient mice was higher than that in control mice (Fig. 2F). Thus, our results demonstrated ARID1A protects mice from virus infection by selectively promoting IFN-I production.

### ARID1A facilitates *Ifn-I* promoters accessibility upon viral infection

**Fig. 3:**
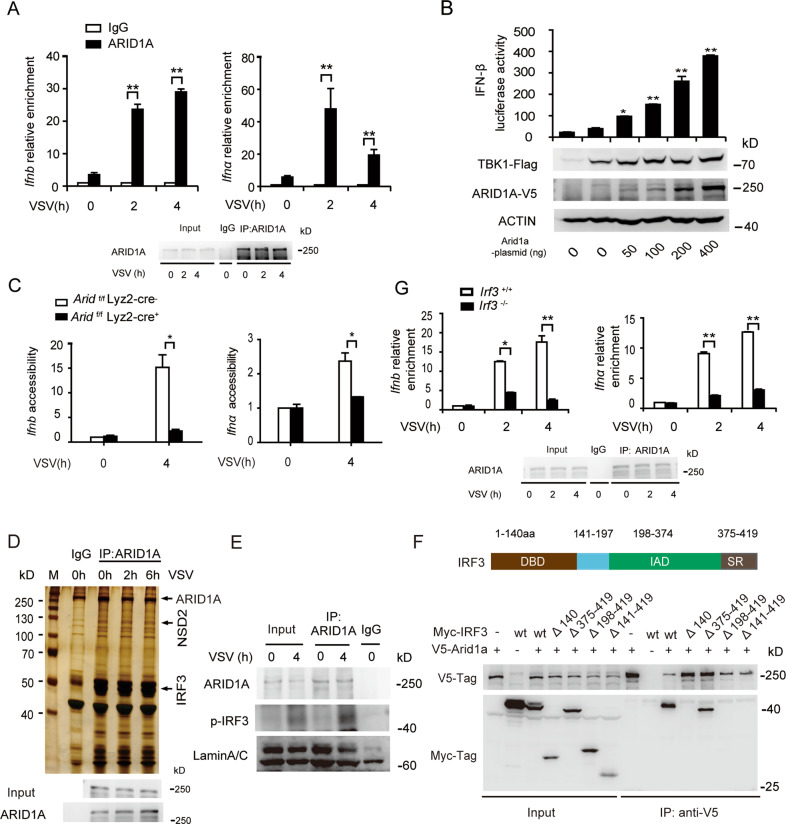
ARID1A is recruited by IRF3 to *Ifn-I* promoters to increase chromatin accessibility. **A** ChIP analysis of ARID1A recruitment to the promoter regions of *Ifn-I* in macrophages infected with VSV (MOI, 1) at indicated times (up panel) and immunoblot analysis of ARID1A expression in ChIP samples (down panel). **B** Luciferase assay of *Ifnb* reporter activity in HEK293T cells transfected with pcDNA3.1-Tbk1-Flag and PEF1a-V5-Arid1a with gradually increased concentrations (upper panel) and immunoblot analysis of ARID1A and TBK1 expression in transfected cells (lower panel). **C** DNA accessibility assay of the *Ifn-I* promoter regions in *Arid1a* ^*f/f*^ Lyz-Cre^*−*^ and *Arid1a* ^*f/f*^ Lyz-Cre^*+*^ BMDMs infected with VSV (MOI, 1) at the indicated times. **D** Silver staining of the proteins immunoprecipitated in the BMDM lysates by ARID1A antibody (up panel) and immunoblot analysis of ARID1A expression in IP samples (down panel). **E** Co-IP analysis of the interaction between endogenous ARID1A and p-IRF3 in the nucleus of BMDMs infected with VSV (MOI, 1) at the indicated times. **F** Co-IP analysis of the interaction between tagged ARID1A and wild type IRF3 or indicated IRF3 mutants in HEK293T cells. **G** ChIP assay of ARID1A recruitment to the promoter regions of *Ifn-I* in *Irf3*^*+/+*^ and *Irf3*^*−/−*^ BMDMs in response to VSV infection (MOI, 1) (up panel) and immunoblot analysis of ARID1A in ChIP samples (down panel). Error bars represent s.d. Student’s *t*-test. *P < 0.05, **P < 0.01. All data are representative of three independent experiments with three biological replicates. (**A**–**C**, **G**: mean ± s.d.).

Given the known function of ARID1A in the mSWI/SNF complex, we performed a chromatin immunoprecipitation (ChIP) assay to examine the association between ARID1A and the promoter region of *Ifnb* and *Ifnα*. We found the recruitment of ARID1A to *Ifn-I* promoter regions gradually increased in BMDMs upon VSV infection (Fig. 3A), suggesting that ARID1A was involved in chromatin remodeling of *Ifn-I* promoter regions thereby affecting their transcriptional activity.

Next, we performed a luciferase reporter assay in HEK293T cells with co-transfection of *Arid1a* and *Tbk1* plasmid. We discovered ARID1A significantly promoted the transcription of *Ifnb* reporter but not *Tnfα* or *Il6* reporter (Fig. 3B, Fig. S6A). Moreover, DNA accessibility of *Ifn-I* promoters was significantly decreased in *Arid1a*-deficient BMDMs compared to control cells (Fig. 3C), but *Arid1a* deletion had no effect on the accessibility of *Il6* or *Tnfα* promoter regions (Fig. S6B). Therefore, our results demonstrated that ARID1A promotes the production of IFN-I by facilitating *Ifn-I* promoter accessibility.

### Viral infection induces IRF3 to bind and recruit ARID1A to *Ifn-I* promoters

Since ARID1A is a sequence non-specific DNA binding protein, its selectivity is probably dependent on the associated protein(s) of ARID1A which could recruit ARID1A to the promoter regions of the given genes [16, 20]. We performed Mass Spectrometry (MS) analysis of VSV infected BMDM lysates subjected to IP with ARID1A antibody and identified IRF3 as a candidate of ARID1A-associated proteins (Fig. 3D; Fig. S6C). Then we confirmed the interaction between endogenous phosphorylated IRF3 with ARID1A in the nucleus of BMDMs upon infection with VSV, but no interaction was observed without VSV infection (Fig. 3E).

Next, immunoprecipitation from HEK293T cells co-transfected with *Arid1a* and truncated *Irf3* plasmids revealed that deletion of the DNA binding domain (DBD; 1-140aa) or the IRF association domain (IAD; 198-374 aa) of IRF3 abrogated its interaction with ARID1A (Fig. 3F). We then performed a ChIP assay with ARID1A antibody in BMDMs derived from *Irf3*-deficient mice and control mice. As expected, the recruitment of ARID1A to the promoter regions of *Ifn-I* was significantly decreased in *Irf3*^*-/-*^ BMDMs in response to VSV infection (Fig. 3G), while absence of IRF3 had no effect on ARID1A recruitment to the promoter regions of *Il6* or *Tnfα* (Fig. S6D). Therefore, our data suggested that ARID1A is selectively recruited by IRF3 to the *Ifn-I* promoters to facilitate chromatin accessibility.

### ARID1A/IRF3 interact with and recruit NSD2 to promote H3K4Me3 and H3K36Me2 of *Ifn-I* promoters

**Fig. 4:**
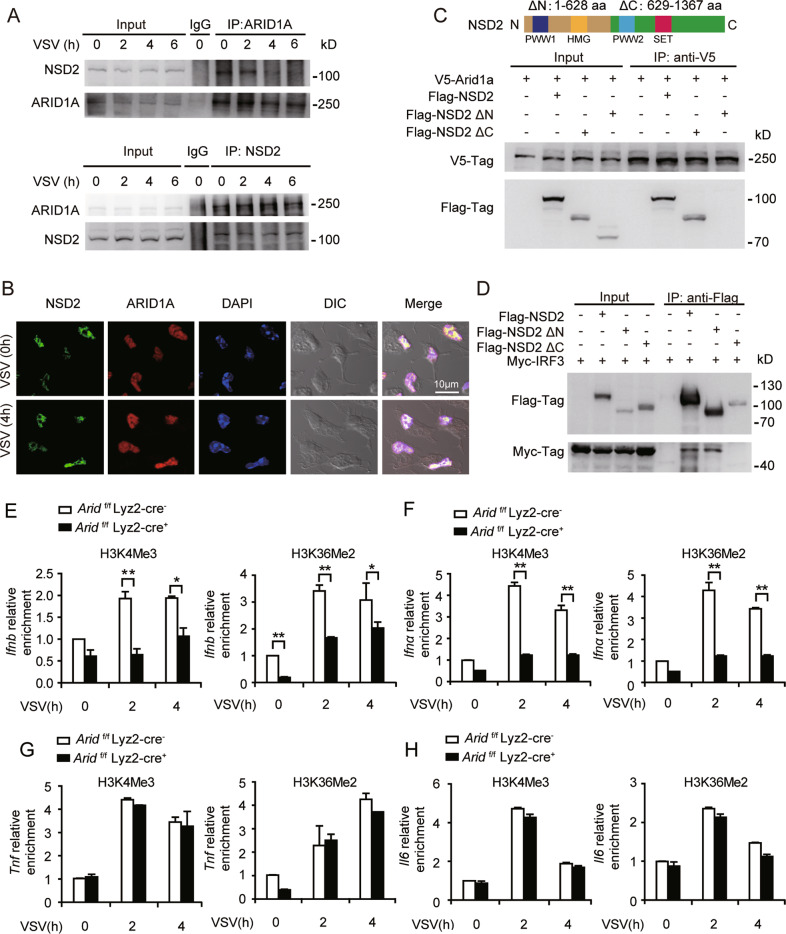
ARID1A interacts with NSD2 to promote methylation of *Ifn-I* promoters. **A** Co-IP analysis of the interaction between endogenous ARID1A and NSD2 in macrophages. **B** Immunofluorescence of ARID1A and NSD2 localization in macrophages (scale bar: 10 μm). **C** Co-IP analysis of the interaction between tagged ARID1A and tagged wild type NSD2 or indicated NSD2 mutants in HEK293T cells. **D** Co-IP analysis of the interaction between tagged IRF3 and tagged wild type NSD2 or indicated NSD2 mutants in HEK293T cells. **E**–**F** ChIP assay of H3K4Me3 and H3K36Me2 in the *Ifnb* (**E**) and *Ifnα* (**F**) promoter regions of *Arid1a* ^*f/f*^ Lyz-Cre^*−*^ and *Arid1a* ^*f/f*^ Lyz-Cre+ BMDMs with VSV infection (MOI, 1) at the indicated times. **G**, **H** ChIP assay of H3K4Me3 and H3K36Me2 in the *Tnfα* (**G**) and *Il6* (**H**) promoter regions of cells as in (**E**–**F**). All data are representative of three independent experiments with three biological replicates. *P < 0.05, **P < 0.01. (**E**–**H**: mean ± s.d.).

Histone modification plays a critical role in regulating DNA accessibility [11, 21], which implies ARID1A might promote DNA accessibility of *Ifn-I* promoter regions through certain histone modification enzymes. By MS analysis, histone methyltransferase NSD2 (Nuclear receptor binding SET domain protein 2) was identified as another ARID1A-associated protein (Fig. 3D; Fig. S6C). The interaction and co-localization between ARID1A and NSD2 were confirmed by both co-IP and immunofluorescence assay (Fig. 4A, B). We further determined that NSD2 interacts with ARID1A by its PWW1 and HMG domains (Fig. 4C). Meanwhile, We found IRF3 could interact with the C-terminal (629-1367aa) of NSD2, indicating that the SET domain-containing region of NSD2 mediates its interaction with IRF3 (Fig. 4D). Therefore, our data suggested ARID1A, IRF3, and NSD2 together form the complex to promote IFN-I production.

NSD2 is widely known as a histone methyltransferase [22], which plays critical roles in regulating DNA accessibility [19]. Therefore, we analyzed histone methylation in the promoter regions of cytokines in response to VSV infection by ChIP assays. We revealed that both H3K4Me3 and H3K36Me2, two types of histone methylation-mediated activators of transcription, at *Ifn-I* promoters were substantially decreased in *Arid1a*-deficient macrophages **(**Fig. 4E, F). Still, *Arid1a*-deficiency did not make any difference in either H3K4Me3 or H3K36Me2 of *Il6* and *Tnfα* promoters **(**Fig. 4G, H). Conclusively, our data demonstrated that ARID1A selectively facilitates chromatin accessibility of *Ifn-I* promoters by recruiting NSD2 to promote H3K4Me3 and H3K36Me2 of *Ifn-I* promoters.

### ARID1A-recruited NSD2 selectively promotes IFN-I production in the antiviral innate response

**Fig. 5:**
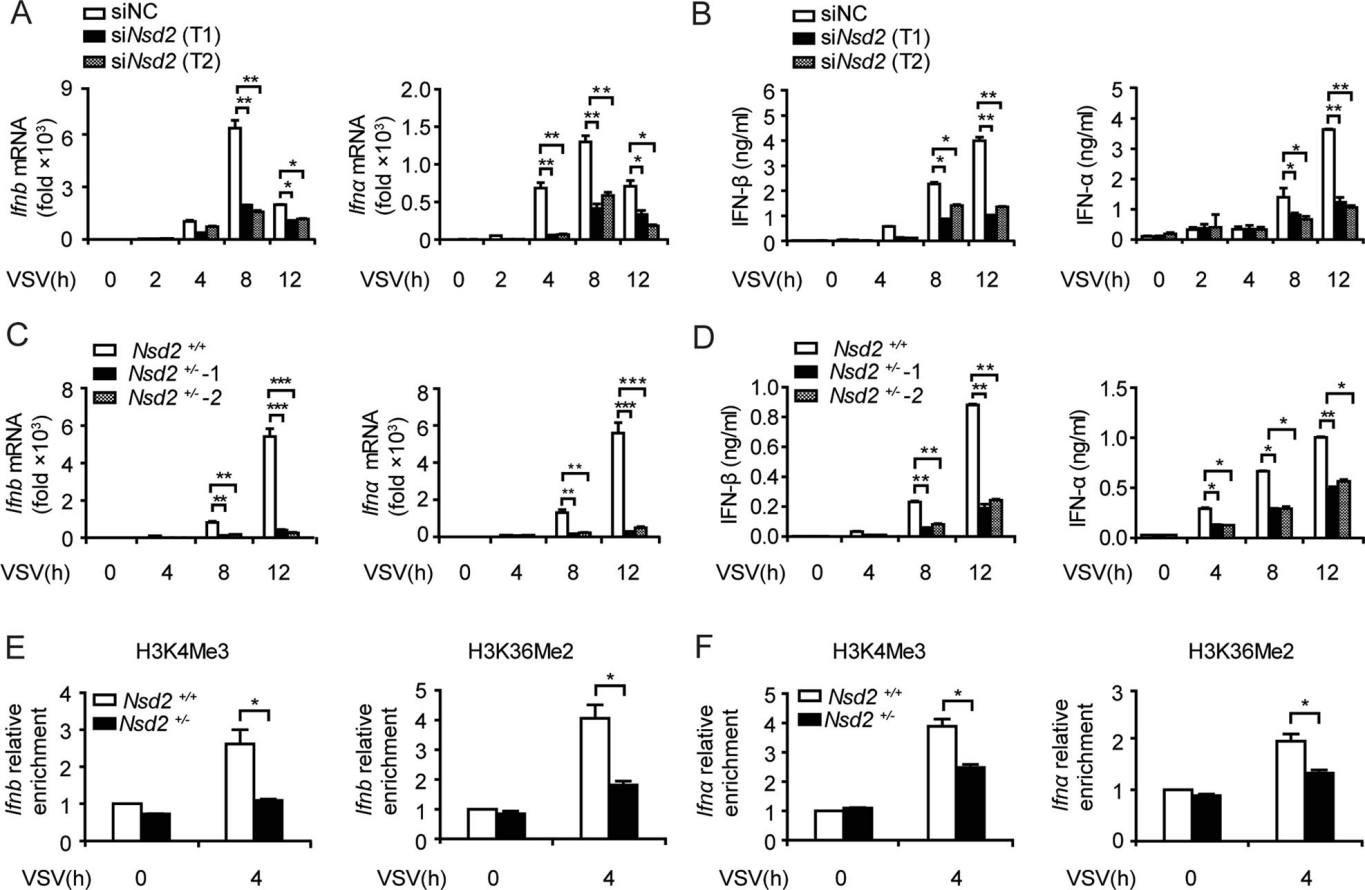
Inhibition of *Nsd2* restrains IFN-I production and histone methylation upon virus infection. **A**, **B** qPCR analysis of *Ifn-I* (**A**) and ELISA analysis of IFN-I (**B**) expression in PMs silenced with two specific *Nsd2* siRNAs or NC siRNA in response to VSV (MOI, 1) infection at the indicated times. **C**, **D** qPCR analysis of *Ifn-I* (**C**) and ELISA analysis of IFN-I (**D**) expression in *Nsd2*^*+/+*^ or *Nsd2*^*+/−*^ RAW 264.7 cells infected with VSV (MOI, 1) at the indicated times. **E**, **F** ChIP assay of H3K4Me3 and H3K36Me2 in the *Ifnb* (**E**) and *Ifnα* (**F**) promoter regions of *Nsd2*^*+/+*^ and *Nsd2*^*+/−*^ RAW264.7 cells infected with VSV (MOI, 1) at the indicated times. *P < 0.05, **P < 0.01. All data are representative of three independent experiments. (**A**–**F**: mean ± s.d.).

We further investigated the function of NSD2 in IFN-I production in response to viral infection. Similarly, we found that the expression of IFN-I, but not IL-6 or TNF-α, was decreased in *Nsd2*-silenced macrophages after VSV infection (Fig. 5A, B; Fig. S7A–C). We attempted to generate *Nsd2* knock-out RAW264.7 cells, but only obtained *Nsd2*^*+/-*^ RAW264.7 cells (Fig. S7D). Consistently, expression of IFN-I (Fig. 5C, D), but not IL-6 or TNF-α (Fig. S7E, F), was decreased in *Nsd2*^*+/-*^ RAW264.7 cells upon VSV infection. Furthermore, both H3K4Me3 and H3K36Me2 levels were decreased in the promoter regions of *Ifn-I* but not of *Il6* or *Tnfα* in *Nsd2*^*+/-*^ macrophages upon VSV infection (Fig. 5E, F; Fig. S7G, H).

**Fig. 6:**
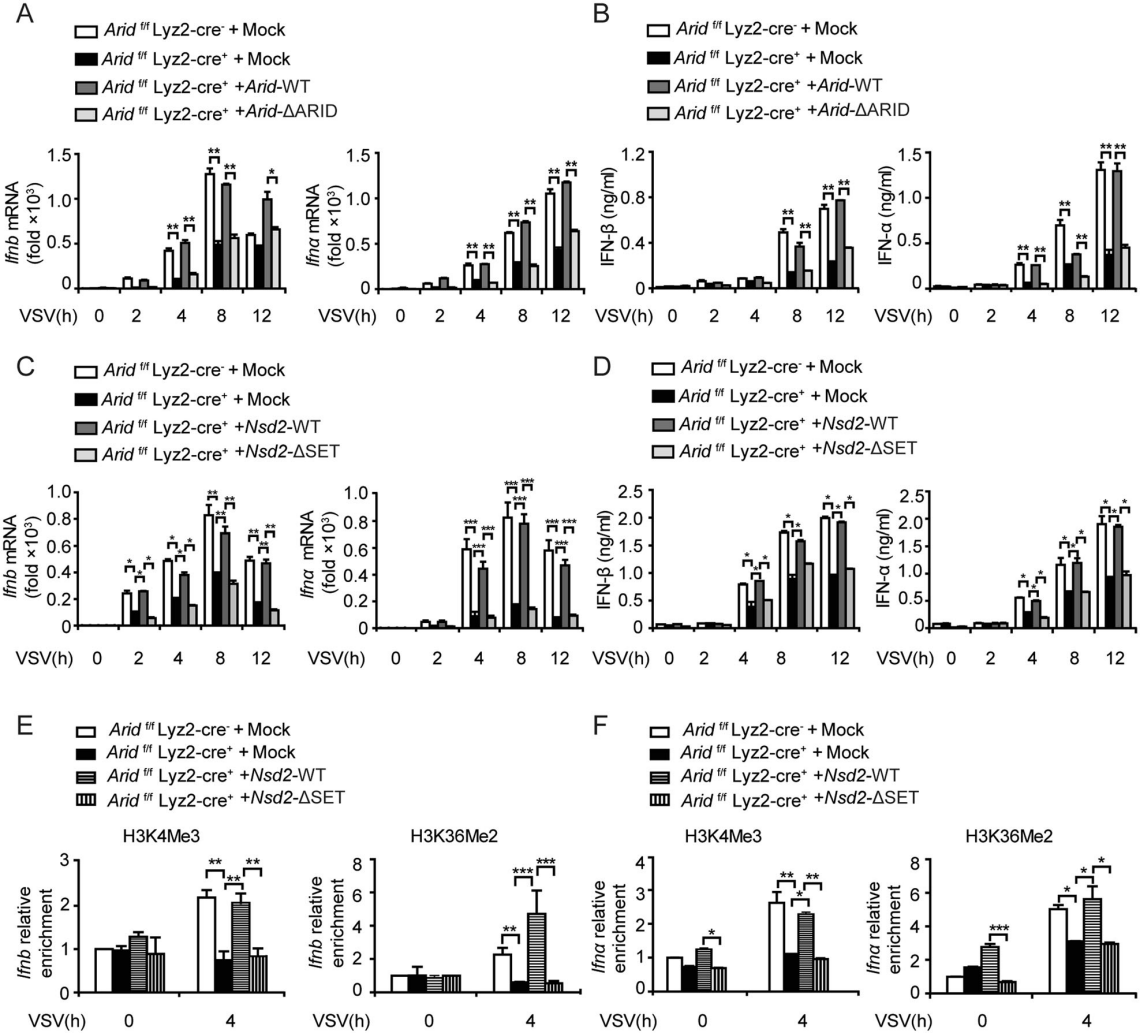
NSD2 promotes IFN-I production by mediating histone methylation of *Ifn-I* promoters. **A, B** qPCR analysis of *Ifn-I* (**A**) and ELISA analysis of IFN-I (**B**) expression in *Arid1a* ^*f/f*^ Lyz-Cre^*-*^ and *Arid1a* ^*f/f*^ Lyz-Cre^*+*^ BMDMs transfected with wild type *Arid1a* and *Arid1a-*ΔARID construct and then infected with VSV (MOI, 1) at the indicated times, mock served as control. **C**, **D** qPCR analysis of *Ifn-I* (**C**) and ELISA analysis of IFN-I (**D**) expression in *Arid1a* ^*f/f*^ Lyz-Cre^*−*^ and *Arid1a* ^*f/f*^ Lyz-Cre^*+*^ BMDMs transfected with wild type *Nsd2* or *Nsd2-*ΔSET construct and then infected with VSV (MOI, 1) at the indicated times, mock served as control. **E**, **F** ChIP assay of H3K4Me3 and H3K36Me2 in the *Ifnb* (**E**) or *Ifnα* (**F**) promoter regions of *Arid1a* ^*f/f*^ Lyz-Cre^*−*^ and *Arid1a* ^*f/f*^ Lyz-Cre^*+*^ BMDMs transfected with plasmids as in (**C**–**D**) in response to VSV (MOI, 1) infection at the indicated times. Error bars represent s.d. Student’s *t*-test. *P < 0.05, **P < 0.01, ***P < 0.001. All data are representative of three independent experiments with three biological replicates. (**A**–**F**: mean ± s.d.).

In addition, transfection of full-length *Arid1a* or *Nsd2* could rescue IFN-I production of *Arid1a*-deficient BMDMs (Fig. 6A–D; Fig. S8A, B) but had no impact on IL-6 or TNF-α production (Fig. S8C, D). Moreover, H3K4Me3 and H3K36Me2 levels of *Ifn-I* promoter regions were recovered after re-expression of wild type ARID1A or NSD2 (Fig. 6E, F). While transfection of either *Arid1a* mutant lacking ARID domain or *Nsd2* mutant missing SET domain failed to restore IFN-I expression to the normal level. Taken together, our results demonstrated that ARID1A recruits NSD2 to mediate H3K4Me3 and H3K36Me2 in the promoter regions of *Ifn-I* thus promotes the production of IFN-I, indicating ARID1A enhances IFN-I expression dependent on its interaction with NSD2.

## Discussion

Production of IFN-I is tightly regulated in the antiviral innate response to effectively eliminate the invading virus but avoid the overproduction-induced tissue damage [23, 24]. Multiple transcription factors including NF-κB, IRF3, IRF7, and AP1 have been reported to promote IFN-I production upon virus infection. Among those transcriptors, IRF3 plays the most critical role in the initiation of IFN-I production [25]. Here we show that ARID1A is recruited by IRF3 to the promoter regions of *Ifn-I*, where it binds histone methyltransferase NSD2 to increase chromatin accessibility and promote IFN-I production in response to virus infection. Therefore, ARID1A binds IRF3 and NSD2 to form a complex in the promoter regions of *Ifn-I* to selectively promote expression of IFN-I, which establishes the crosstalk between histone modification and chromatin remodeling in transcriptional regulation of antiviral gene expression.

Previous reports showed that the mSWI/SNF complex is recruited to the promoter of *Ifnb* to force nucleosome displacement from the transcription start site [15, 26, 27]. However, the sequential recruitment of ATP-dependent remodelers and other modifiers to the promoter regions is still controversial. Also, whether the mSWI/SNF complex or specific components might participate in the regulation of interferon production remains unclear to date. ARID1A, which mediates the location of the mSWI/SNF complex by its DNA-binding domain, is not essential for the ATP-dependent chromatin remodeling function of the complex [16, 28]. Recent studies showed that ARID1A mediates the interaction between mSWI/SNF complex and YAP/TAZ thus involves in carcinogenesis and liver regeneration [29, 30]. Our results indicated that ARID1A is not only a subunit of mSWI/SNF complex that helps to target specific regions of the genome, but also selectively and epigenetically promotes antiviral IFN-I production. On the one side, we found ARID1A interacts with IRF3, a crucial transcription factor that regulates the production of IFN-I in response to virus infection [31, 32]. IRF3 could recruit ARID1A to the promoter sites of *Ifn-I* which could explain ARID1A involves specific regulation of IFN-I but not other cytokines production. On the other hand, ARID1A also binds with methyltransferase NSD2 to change the chromatin accessibility of *Ifn-I* promoter regions by histone modification.

Histone acetylation has a critical role in the regulation of IFN-I production during the innate response against viruses [33]. However, histone methylation has been rarely recognized as a mechanism of transcriptionally regulating IFN-I production [3, 34]. As one of the most important methyltransferase groups, the NSD family could monomethylate and dimethylate histone H3 on lysine 36 (H3K36) [35]. Besides, the NSD family also targets the non-histone protein. For example, NSD1 or NSD3 could promote antiviral innate response by mediating p65 methylation at lysine residues K218 and K221 [36], or promoting IRF3 methylation at lysine residue K366 respectively [37]. Also, NSD2 was reported to interact with histone chaperone SPT6 and facilitate interferon-induced transcription [38]. Here, we illustrated NSD2 could interact with ARID1A to promote antiviral immune response by methylating H3K4Me3 and H3K36Me2, thus increasing chromatin accessibility in the promoter regions of *Ifn-I*. Our results suggested IRF3, ARID1A and NSD2 together compose the complex at the promoters of *Ifn-I* to cooperate in the regulation of IFN-1 expression. Further work is needed to clarify the exact association inside the IRF3-ARID1A-NSD2 complex and evaluate the potential interactions between the IRF3-ARID1A-NSD2 complex and the mSWI/SNF complex. In addition, the question of whether there would be other protein regulators involved in the above-mentioned complex or whether this complex might target to other possible genes is yet to be answered.

Pathogens have evolved a variety of epigenetic strategies for their survival and replication, such as induction of host protein and chromatin modifications to inhibit the expression of activators and enhance the expression of repressors in innate immunity [39, 40]. The analyses of the GEO database showed the decreased *Arid1a* mRNA expression in hepatitis C patients (GSE38597), gastric cancer infected with Epstein Barr virus [41], epithelial cell model of kaposarcoma virus infection (GSE1377), and cervical cancer infected with human papillomavirus [42]. These results indicated that in chronic viral infection, especially along with carcinogenesis, *Arid1a* expression may be downregulated and involved in the immune escape and pathogenesis. In the current study, we utilized virus-infected macrophages as the cell model and found that the protein expression of ARID1A remains unchanged in these cells in response to acute infection with RNA or DNA viruses. The results indicated that ARID1A may have different responses to acute and chronic viral infections. More studies are further needed to explore the expression pattern and roles of ARID1A protein in infectious diseases and virus infection-related cancers.

In conclusion, our study provides a mechanistic insight into the epigenetic regulation of antiviral innate immune response by integrating histone modification with chromatin remodeling. Our study may provide a potential target for the control of virus infectious diseases and other inflammatory diseases.

## Materials and methods

### Mice

Animal experiments involved in this study were performed with the guidelines of the Institutional Animal Care, and the experimental design and process were approved by the animal ethics review (ACUC-A01-2018-018). The *Arid1a* floxed mice [17] and lyz2-cre mice (004781, the Jackson Laboratory) were bred to induce deletions between the two loxP at exon 9-11 of the *Arid1a* gene. *Arid1a* ^*f/f*^ Lyz-Cre^+^ mice were defined as *Aird1a*-deficient mice, *Arid1a* ^*f/f*^ Lyz-Cre^-^ littermate mice were used as control. *Irf3*-deficient mice were provided by Dr. T. Taniguchi from the University of Tokyo, Tokyo, Japan. All mice were genotyped by PCR before experimentation. The genotyping primers were *Arid1a* forward, 5’- TAAGTTCCAAGACAAGCAGAACC- 3’; *Arid1a* reverse, 5’- ACAGAATGGAGTGAAGACACGAT- 3’; *lyz2*- wt: 5’- TTACAGTCGGCCAGGCTGAC- 3’; *lyz2*-common: 5’- CTTGGGCTGCCAGAATTTCTC- 3’; *lyz2*-mutation: 5’- CCC AGAAATGCCAGATTACG- 3’.

### Cell culture

PMs were obtained from mice 3 days after intraperitoneal injection with thioglycolate, BMDMs were prepared by culturing in recombinant 50 ng/ml mouse M-CSF (Perprotech) for 7 days. HEK293T, RAW264.7, and Vero cells were obtained from American Type Culture Collection. Cells were all cultured in DMEM supplemented with 10% FBS.

### Pathogens

VSV (Indiana strain) and HSV-1 were amplified from Vero cells and SeV (Sendai virus) was amplified from chicken embryos as described previously [43].

### RNA interference

siRNAs of *Arid1a* (Invitrogen) and siRNAs of *Nsd2* (Genepharma) and RNAi MAX reagent (Invitrogen) were utilized for RNA interference in PMs, and the interference efficiency was examined by qPCR. Sequences of siRNAs were listed as follows: *Arid1a* sense1, 5’-AGAUGUGGGUGGACCGGUATT-3’; *Arid1a* sense2, 5’-GAAUGAACAGGAAAACUCATT-3’; *Nsd2* sense1, 5’-GCA AAGCUCAACUUUCUAATT-3’; *Nsd2* sense2, 5’-GCA GAAAUUUAAUGGCCAUTT-3’. The detected primers for efficiency of RNA interference are: si *Arid1a* forward, 5’- GAACCCTATGGGTGCTGGAG-3’; si *Arid1a* reverse, 5’-CCCATCATGCCCCCTTGATT-3’; si *Nsd2* forward, 5’- GGCCAGAACAAGCTCTTACAA-3’; si *Nsd2* reverse, 5’-TGTGGGCTCCCATAA AAGCTC-3’.

### In vivo animal experiments

The mice were infected with VSV by tail vein injection (5 × 10^7^ pfu/g). Serum was collected for ELISA detection at indicated times. The RNA of the spleen, liver, lung, and PM cells were extracted by TRIzol (Invitrogen). The titer of VSV in cell lysates (0.1%Triton in PBS) of mouse organs was detected by TCID50. The lung lobes of mice were fixed with 4% paraformaldehyde. After embedding, sections were stained with hematoxylin and eosin, and observed with Pannoramic SCAN. For survival curve analysis, mice were intravenously injected with VSV (1 × 10^8^ pfu/g), then generated and analyzed for statistical significance with GraphPad Prism 5.0.

### Quantitative real-time PCR assay

Total RNA was extracted from cells and animal tissues using TRIzol reagent (Ambion) or RNAfast2000 kit (Fastagen). ReverTra Ace qPCR RT master mix (Toyobo) was used to synthesize cDNA.Quantitative PCR analysis was performed by Q7 (ABI) using the SYBR RT-PCR kit (TOYOBO). Sequences of the primers for qPCR were listed as follows: *Ifnb* forward, 5’-ATGAGTGGTGGTTGCAGGC-3’; *Ifnb* reverse, 5’- TGACCTTTCAAATGCAGTAGA-3’; *Ifnα4* forward, 5’- TACTCAGCAGACCTTGAACCT; *Ifnα4* reverse, 5’-CAGTCTTGG CAGCAAGTTGAC-3’; *Tnf* forward, 5’-AAGCCTGTAGCCCACGTCGTA-3’; *Tnf* reverse, 5’-GGCACCACTAGTTGGTTGTCTTTG-3’; *Il6*- forward, 5’- TAG TCCTTCCTACCCCAATTTCC-3’; *Il6* reverse, 5’- TTGGTCCTTAGCCACTCC TTC-3’; *Isg15* forward, 5’- GGTGTCCGTGACTAACTCCAT-3’; *Isg15* reverse, 5’-TGGAAAGGGTAAGACCGTCCT-3’; *Ifit1* forward, 5’-CTGAGATGTCAC TTCACATGGAA-3’; *Ifit1* reverse, 5’-GTGCATCCCCAATGGGTTCT-3’; *Ccl5* forward, 5’-GCTGCTTTGCCTACCTCTCC-3’; *Ccl5* reverse, 5’-TCGAGTGAC AAACACGACTGC-3’; *Cxcl10* forward, 5’- CCAAGTGCTGCCGTCATTTTC-3’; *Cxcl10* reverse, 5’- GGCTCGCAGGGATGATTTCAA-3’.

### Immunofluorescence

PMs plated on glass coverslips were infected with or without VSV for 8 hours and labeled with antibodies. Antibody to ARID1A (32761) was from Santa Cruz. Antibody to NSD2 (A7938) was from ABclonal. Antibody to Alexa488 (A1108) and Alexa594 (A11005) were from Invitrogen. Slides were subjected to microscopy analysis with Zeiss LSM780 confocal laser microscope.

### Plasmid constructs and transfection of cells

Expression vectors encoding ARID1A were obtained from Origene (MR224275) and cloned into PEF1a-V5-vector. FLAG-tagged-NSD2, FLAG-tagged-TBK1, Myc-tagged IRF3, and their mutants were cloned into pcDNA3.1 vector. All constructs were confirmed by DNA sequencing. HEK 293T cells were transfected with JetPEI reagents (PolyPlus). RAW246.7 cells were transfected with FuGENE HD (Promega), and BMDMs were transfected with Lipofectamine 3000 (Thermo) according to the manufacturer’s instructions.

### Immunoblot and immunoprecipitation

Immunoblot and immunoprecipitation assays were performed as described previously [44]. Cytoplasmic and nuclear proteins were separated using MinuteTM Cytoplasmic and Nuclear Extraction Kit (SM-003, Invent). Antibodies to V5-Tag (13202 S), Lamin A/C (4C11) (4777 S) were from Cell Signaling Technology. Antibodies to Myc-tag (M192-3), GAPDH (M171-3), and β-Actin (M177-3) were from MBL.

### ELISA

ELISA kits (Biolegend and Ebioscience) were used for examining the concentrations of cytokines in cell supernatants or serum of mice according to the manufacturer’s instructions.

### Flow cytometry assay

Cell preparations and surface staining were performed as described previously [44]. The data were analyzed by BD FACS Diva software (BD Biosciences). Antibody to Siglec-F (562681) was from BD Biosciences. Antibodies to CD45 (103108), CD3 (100220), CD19 (115529), CD45 (103155), CD11b (101228), F4/80 (123109), NK-1.1 (108707), Ly-6C (128024), CD4 (100412), CD8a (100712), CD11c (117307), I-A/I-E (107629) and CD49b (108907) were from BioLegend.

### RNA-seq detection and analysis

Total RNAs were isolated with TRIzol. Then RNA quantification and qualification were confirmed. Library construction and sequencing were performed on Illumina Novaseq 6000 (Novegene, http://www.novogene.com/). Gene FPKMs were computed by summing the FPKMs of transcripts in each gene group. Transcripts with a *P*-adjust < 0.05 were assigned as differentially expressed. KOBAS software was used to test the statistical enrichment of differentially expressed genes in KEGG pathways.

### DNase I sensitivity assay

DNase I sensitivity assay was performed as described previously [45]. In brief, the cell precipitation was digested with (5 U/μl) Dnase I enzyme and conducted with 0.5 M EDTA. DNA was extracted by the phenol-chloroform method. Chromatin accessibility of the promoter regions was analyzed by qPCR. Changed fold was concluded using 2ΔCt with respect to *Arid1a (f/f)*-0h set to a value of 1. Sequences of the primers for qPCR are listed as follows: *Ifnb* promoter forward, 5’-TAACCCAGTACATAGCATATA- 3’; *Ifnb* promoter reverse, 5’-AGTGAGAATGATCTTCCTTCAT- 3’; *Ifnα4* promoter forward, 5’-ATCCCA GACACAAGCAGAGAG- 3’; *Ifnα4* promoter reverse, 5’-GGCTGTGGGTTTGAG TCTTCT- 3’; *Tnf* promoter forward, 5’-CAGCCACTGCTTGGCTAGAC; *Tnf* promoter reverse, 5’-CGGATCCCATGGACCAACTG- 3’; *Il6* promoter forward, 5’-GCAGTGGGATCAGCACTAAC- 3’; *Il6* promoter reverse, 5’-GGTGGGTAA AGTGGGTGAAG-3’.

### ChIP-PCR

The cells were treated as the instructions of the ChIP assay kit (Beyotime P2078). For each ChIP assay, antibodies were added to cell samples and incubated overnight at 4 °C. Antibody–chromatin complexes were pulled down by magnetic protein G beads (Invitrogen). DNA was eluted, purified, and subjected to qPCR. Antibodies to ARID1A (ab182560) and H3K4Me3 (ab8580) were from Abcam. Antibody to H3K36Me2 (39255) was from Active Motif. Sequences of the primers for qPCR are the same as the primers in DNase I Sensitivity Assay.

### CRISPR-Cas9-mediated knockout RAW264.7 cells

*Arid1a*^*_/−*^ and *Nsd2*^*+/−*^ cells were generated by CRISPR/Cas9 system in the RAW264.7 cell line. Briefly, the *Arid1a*^_/_^ RAW264.7 cells were transfected with Cas9 and pGL3-U6-sgRNA plasmid, then added blasticidin S hydrochloride (2 μg/mL; Life Technology) and puromycin (3 μg/mL; Life Technology) after 24 h. *Nsd2*^*+/−*^ RAW264.7 cells were transfected with sgRNAs–targeted-Cas9/green fluorescent protein (PGX458) plasmid. Single transfected cells were sorted. The clones were detected by PCR. The sequence of sgRNA are listed as follows: *Arid1a*- A1 up: 5’-CACCCTGGTTATAGTATGGAGTC-3’; *Arid1a* -B1 up: 5’-CACCCTGTTGTGTGGTGGACTGC-3’; *Nsd2*-AW1 up: 5’-CACCAAATCCTTGGCAGTGCAAA-3’; *Nsd2*- BW1 up: 5’- CACCACTAGGAGGAACAGGAAG-3’.

### Assays of dual-luciferase reporter gene expression

For reporter assays, HEK293T cells were co-transfected with the reporter plasmid, pRL-TK-Renilla-luciferase plasmid, and other plasmids according to the experiment design and measured with the Dual-Luciferase Reporter Assay System Kit (Promega) according to the manufacturer’s protocols.

### Statistical analysis

Statistical significance between groups was determined by a two-tailed Student’s *t*-test. *P < 0.05 were considered to be significant (**P < 0.01, ***P < 0.001). For the mouse survival study, Kaplan–Meier survival curves were generated and analyzed for statistical significance with GraphPad Prism 5.0.

## Supplementary information

Supplementary Material

Supplementary Material

Supplementary Material

Supplementary Material

Supplementary Material

Supplementary Material

Supplementary Material

Supplementary Material

Supplementary Material
