# Supplementary material for: Chromatin remodeler ARID1A binds IRF3 to selectively induce antiviral interferon production in macrophages
Source: Cell Death Dis. 2021 Jul 27;12(8):743. doi: 10.1038/s41419-021-04032-9 (PMC8316351; doi:10.1038/s41419-021-04032-9)
Supplement: Supplementary file 1 — Supplementary Material [file 41419_2021_4032_MOESM1_ESM.docx]

**Supplementary Information**

**Fig. S1. ARID1A is located in the nucleus and remains unchanged in response to different virus infection.** (*A*) qPCR analysis of *Arid1a* expression in multiple mice organs. (*B*) Immunofluorescence analysis of endogenous ARID1A localization in macrophages (scale bar: 10μm). (*C*) Immunoblot analysis of ARID1A expression in separated nuclear and cytoplasmic compartments. (*D-F)* Immunoblot analysis of ARID1A expression in PMs (*D*), BMDMs (*E*) and RAW264.7 cells (*F*) infected with VSV (MOI, 1), SeV (MOI, 1) and HSV-1 (MOI, 10) at the indicated times. (*G*) Immunoblot analysis of ARID1A expression in splenocytes of mice injected with VSV (5 × 10^7^ pfu/g, n=3 per group) by tail vein at the indicated times. All data are representative of three independent experiments with three biological replicates. (*A*: mean±s.d.).

**Fig. S2. Inhibition of ARID1A has no impact on production of TNF-α and IL-6 upon VSV infection.** (*A*) qPCR analysis of silencing efficiency in PMs transfected with *Arid1a*-specific siRNAs and control siRNA. (*B, C*) qPCR (*B*) and ELISA (*C*) analysis of TNF-α and IL-6 expression in *Arid1a*-silenced PMs in response to VSV (MOI, 1) infection at the indicated times. (*D*) Schematic illustrating the generation of *Arid1a* knockout RAW264.7 cells and PCR analysis of *Arid1a* deletion. (*E, F*) qPCR (*E*) and ELISA (*F*) analysis of TNF-α and IL-6 expression in *Arid1a* knock-out RAW264.7 and control cells infected with VSV (MOI, 1) at the indicated times. (*G*) qPCR analysis of *Ifit1, Cxcl10, Ccl5* and *Mx1* expression in the cells as (*E-F*). (*H*) Immunoblot analysis of ARID1A expression in RAW264.7 cells transfected with PEF1a-V5-Arid1a. (*I, J*) qPCR analysis of *Ifn-I* (I) and ELISA analysis of IFN-I (*J*) expression in *Arid1a* overexpressed RAW264.7 and control cells infected with VSV (MOI, 1) at the indicated times. Error bars represent s.d. Student’s t-test. *P＜0.05, **P＜0.01. All data are representative of three independent experiments with three biological replicates. (A-C, E-G, I-J: mean±s.d.).

**Fig. S3. Deficiency of ARID1A does not affect the production of TNF-α and IL-6 upon VSV infection.** (*A-B*) qPCR analysis of *Arid1a* gene deletion (*A*) and immunoblot of ARID1A expression (*B*) in BMDMs from *Arid1a* *^f/f^* Lyz-Cre*^-^* and *Arid1a* *^f/f^* Lyz-Cre*^+^* mice. (*C*) Co-IP analysis of the interaction between endogenous BRG1 and ARID1A, BAF170, BAF155 in BMDMs of *Arid1a* *^f/f^* Lyz-Cre*^-^* and *Arid1a* *^f/f^* Lyz-Cre*^+^* mice infected with VSV (MOI, 1) at the indicated times. (*D*) qPCR analysis of *Ifit1, Cxcl10, Ccl5* and *Mx1* expression in *Arid1a*-deficient BMDMs and control cells in response to VSV (MOI, 1) at the indicated times. (*E-J*) qPCR (*E, G, I*) and ELISA (*F, H, G*) analysis of TNF-α and IL-6 expression in *Arid1a*-deficient BMDMs and control cells in response to VSV (MOI, 1) (*E, F*), SeV (MOI, 1) (*G, H*) and HSV-1 (MOI, 10) (*I, J*) infection at the indicated times. Error bars represent s.d. Student’s t-test. *P＜0.05, **P＜0.01. All data are representative of three independent experiments with three biological replicates. (*A-J*: mean±s.d.).

**Fig. S4. Deficiency of ARID1A interferes with IFN-I signaling pathway by RNA-Seq analysis.** (*A*) The cluster heat map of differentiated genes in *Arid1a* *^f/f^* Lyz-Cre*^-^* and *Arid1a* *^f/f^* Lyz-Cre*^+^* BMDMs without or with VSV (MOI, 1) infection for 8 hrs. (*B*) The cluster heat map of decreased interferon genes involved in the immune response. (*C, D*) KEGG (Kyoto Encyclopedia of Genes and Genomes) analysis of data in (*A*) involved in signaling pathways.

**Fig. S5. Deficiency of ARID1A has no effect on the development of immune cells.** (*A*) Flow cytometry analysis of CD45^+^ cells, including CD4^+^ T cells, CD8^+^ T cells, B cells, macrophages, dendritic cells, NK cells, neutrophils and eosinophils in spleen of *Arid1a* *^f/f^* Lyz-Cre*^-^* and *Arid1a* *^f/f^* Lyz-Cre*^+^* mice. (*B*) Statistical analysis of the ratio of immune cells in (*A*). (*C*) The numbers of BMDMs (left) and PMs (right) from *Arid1a*-deficient and control mice. Error bars represent s.d. Student’s t-test. All data are representative of three independent experiments with three biological replicates. (*B, C*: mean±s.d.).

**Fig. S6. NSD2 and IRF3 are identified as ARID1A associated proteins by MS analysis.** (*A*) Luciferase activity assay of *Tnfα and Il6* reporter activity in HEK293T cells transfected with PEF1a-V5-Arid1a in response to VSV (MOI, 1) infection at the indicated time. (*B*) DNA accessibility assay of *Tnfα* and *Il6* promoter regions of *Arid1a* *^f/f^* Lyz-Cre*^-^* and *Arid1a* *^f/f^* Lyz-Cre*^+^* BMDMs with VSV (MOI, 1) infection at the indicated times. (*C*) Peptides of IRF3 and NSD2 immunoprecipitated with ARID1A antibody. (*D*) ChIP assay of ARID1A recruitment to the promoter regions of *Tnfα* and *Il6* in *Irf3^+/+^* and *Irf3^-/-^* macrophages in response to VSV (MOI, 1) at the indicated times. Error bars represent s.d. Student’s t test. All data are representative of three independent experiments with three biological replicates. (*A, B and D*: mean±s.d.).

**Fig. S7. NSD2 does not impair TNF-α and IL-6 productions upon VSV infection.** (*A*) qPCR analysis of silencing efficiency in PMs transfected with *Nsd2* specific and control siRNA. (*B, C*) qPCR (B) and ELISA (*C*) analysis of TNF-α and IL-6 expression in the cells as (*A*) (*D*) Schematic illustrating the generation of *Nsd2* knockout RAW264.7 cells and PCR analysis of *Nsd2* gene deletion. (*E, F*) qPCR (*E*) and ELISA (*F*) analysis of TNF-α and IL-6 expression in *Nsd2^+/+^* and *Nsd2^+/-^* RAW264.7 infected with VSV (MOI, 1) at the indicated times. (*G, H*) ChIP assay of the *Tnfα* (*G*) and *Il6* (*H*) promoter regions in *Nsd2^+/+^* and *Nsd2^+/-^* RAW264.7 infected with VSV (MOI, 1) for 4 hrs. Error bars represent s.d. Student’s t test. All data are representative of three independent experiments with three biological replicates. (*A-H*: mean±s.d.).

**Fig. S8. ARID1A and NSD2 have no function on TNF-α and IL-6 production upon VSV infection.** (*A, B*) qPCR analysis of *Arid1a* *^f/f^* Lyz-Cre*^-^* and *Arid1a* *^f/f^* Lyz-Cre*^+^* BMDMs transfected with wild type *Arid1a* and *Arid1a*-ΔARID construct (*A*) or with wild type *Nsd2* and *Nsd2-*ΔSET construct (*B*), mock served as control. (*C, D*) qPCR (*C*) and ELISA (*D*) analysis of TNF-α and IL-6 expression in the cells as (*B*) with VSV (MOI, 1) infection at the indicated times, mock served as control. Error bars represent s.d. Student’s t-test. All data are representative of three independent experiments with three biological replicates. (*A-D*: mean±s.d.).
