# Supplementary figures and images for: Chromatin remodeler ARID1A binds IRF3 to selectively induce antiviral interferon production in macrophages
Source: Cell Death Dis. 2021 Jul 27;12(8):743. doi: 10.1038/s41419-021-04032-9 (PMC8316351; doi:10.1038/s41419-021-04032-9)

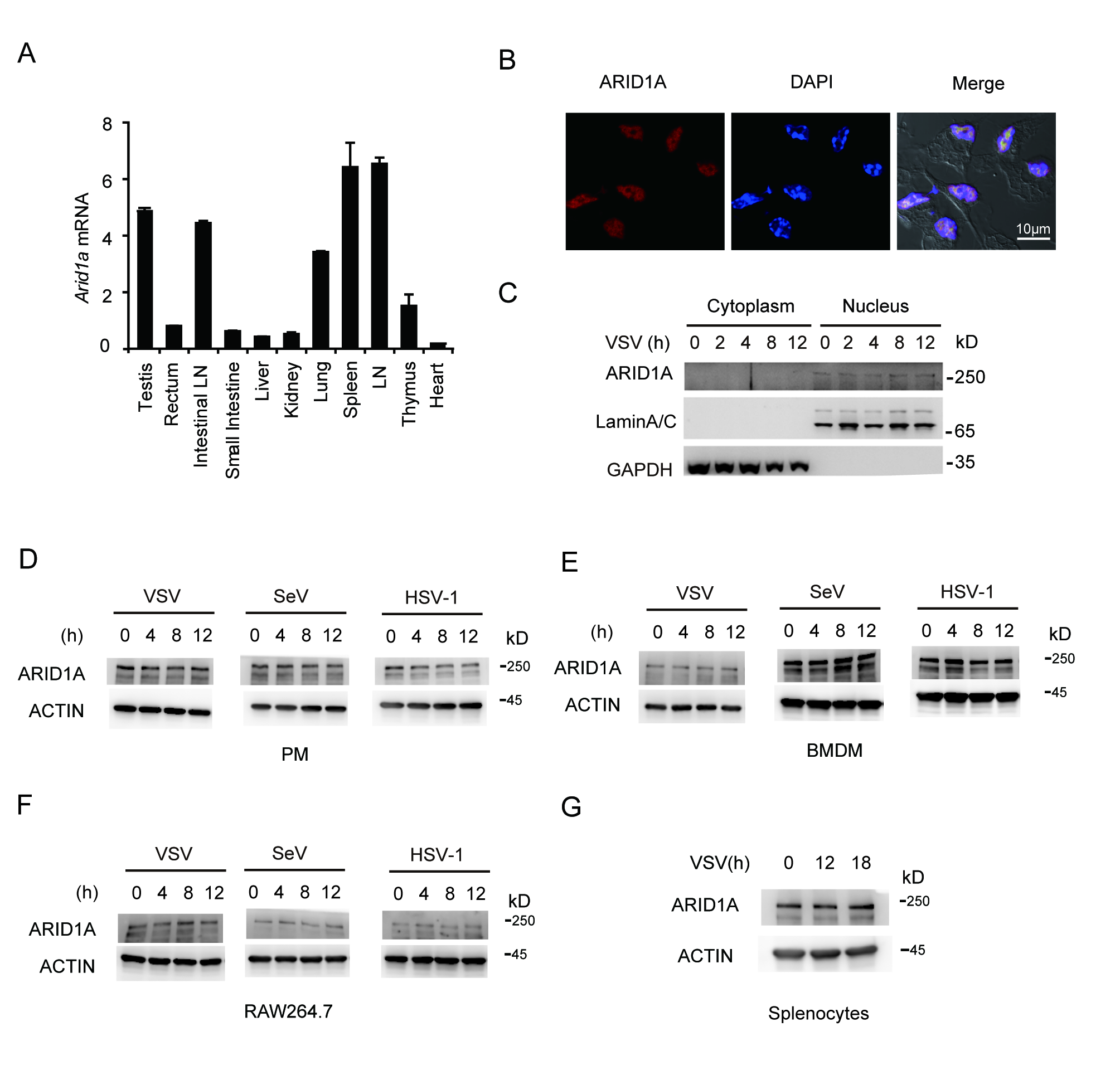

Supplement: Supplementary file 2 — Supplementary Material [file 41419_2021_4032_MOESM2_ESM.tif]

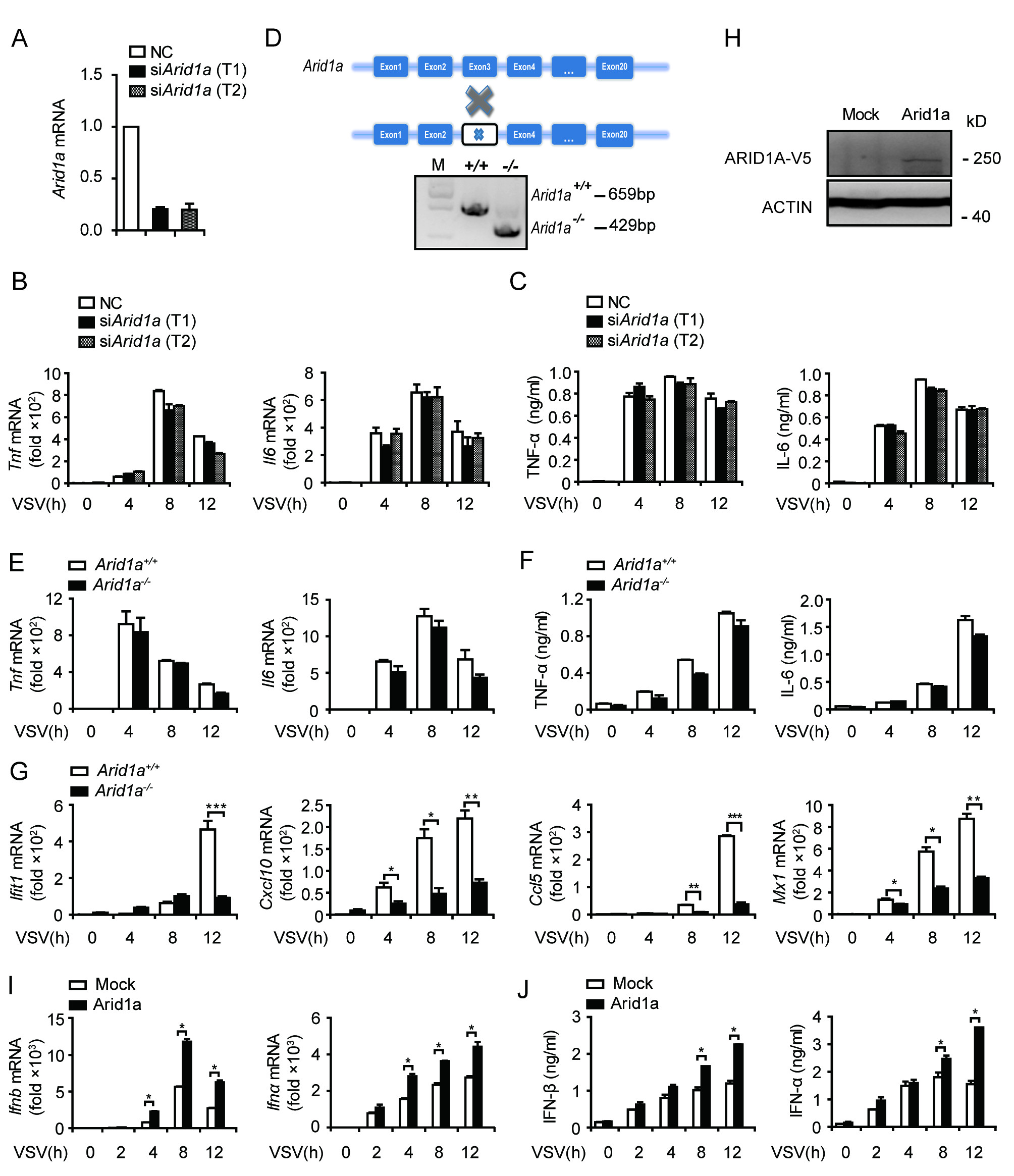

Supplement: Supplementary file 3 — Supplementary Material [file 41419_2021_4032_MOESM3_ESM.tif]

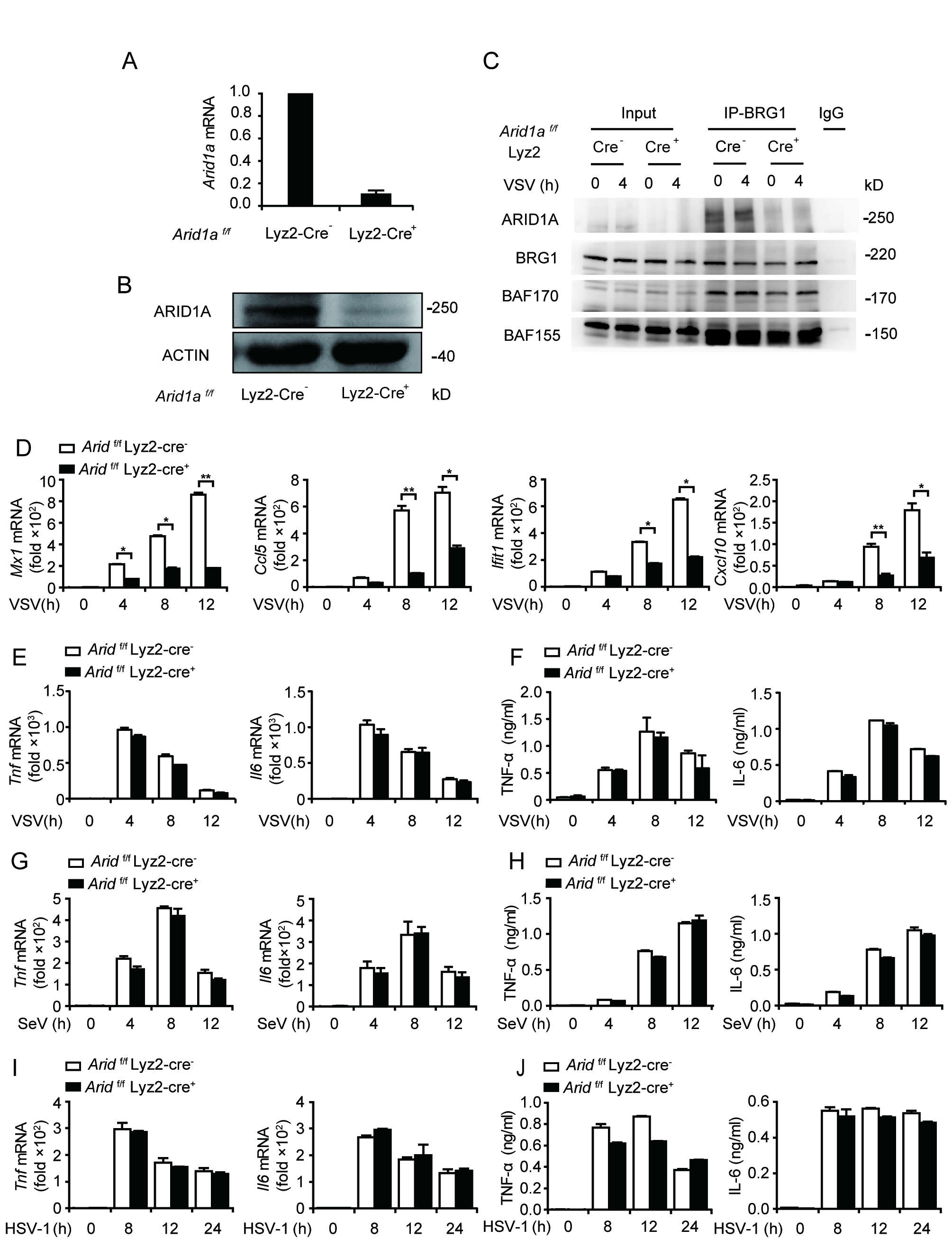

Supplement: Supplementary file 4 — Supplementary Material [file 41419_2021_4032_MOESM4_ESM.tif]

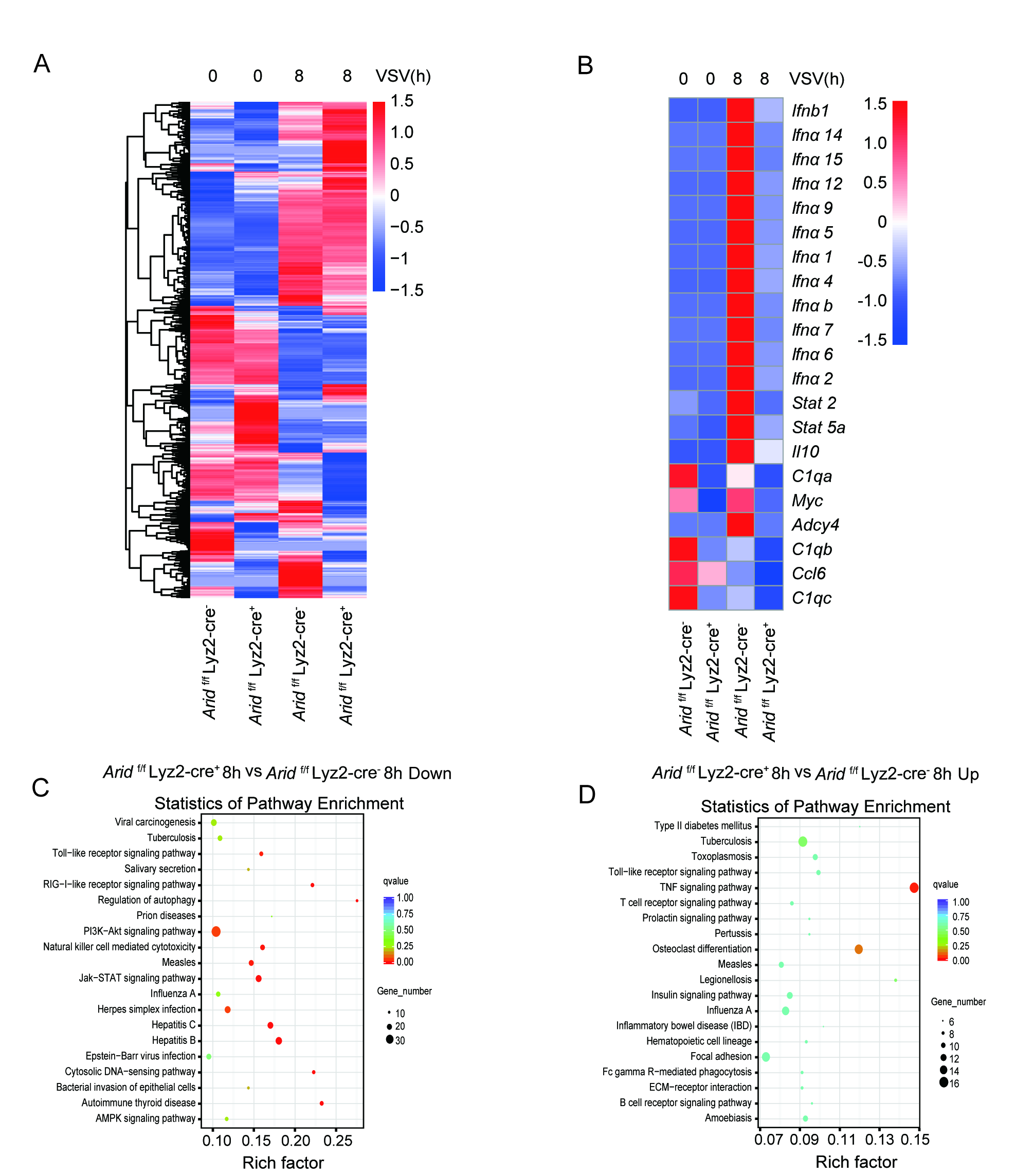

Supplement: Supplementary file 5 — Supplementary Material [file 41419_2021_4032_MOESM5_ESM.tif]

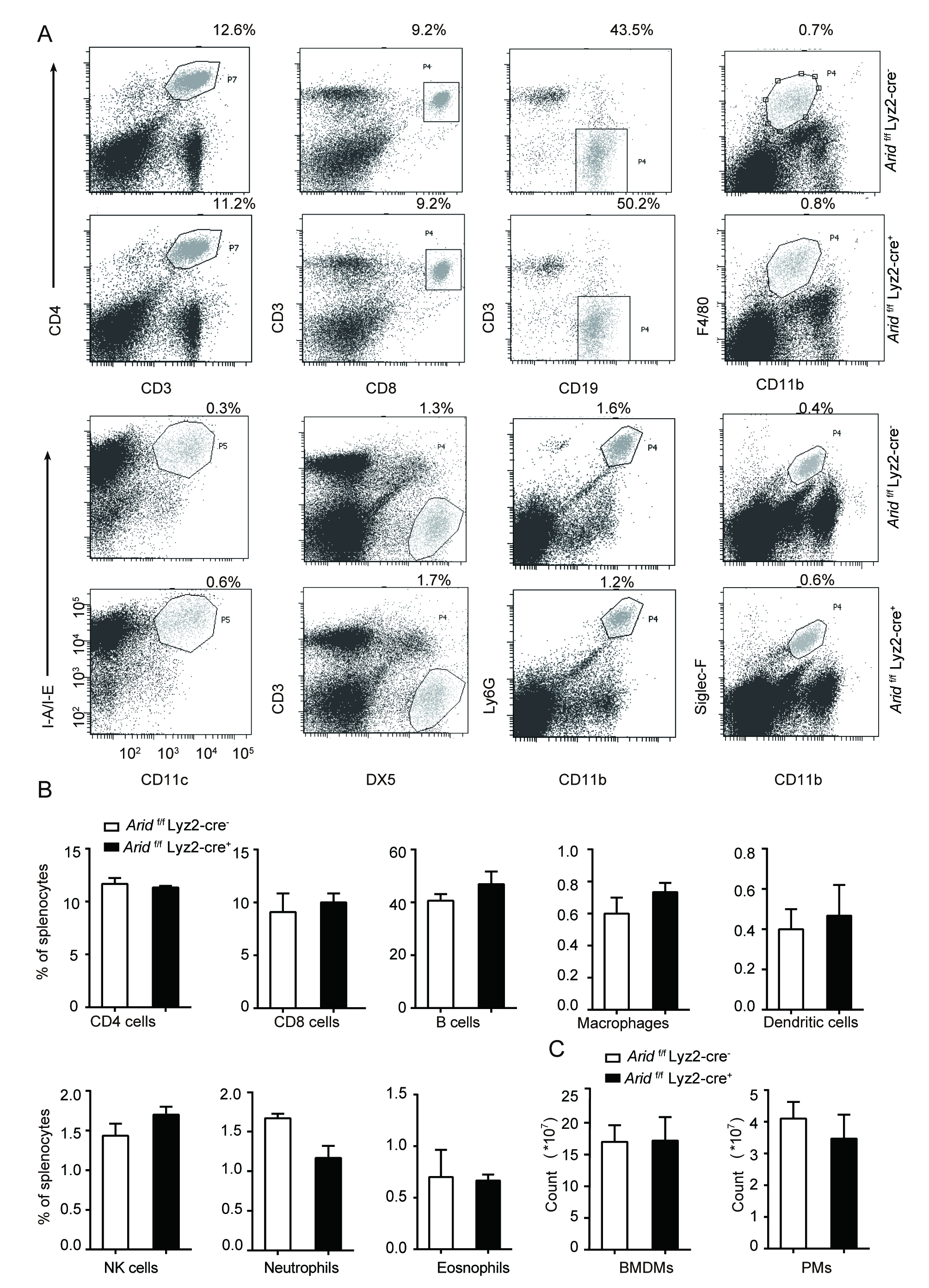

Supplement: Supplementary file 6 — Supplementary Material [file 41419_2021_4032_MOESM6_ESM.tif]

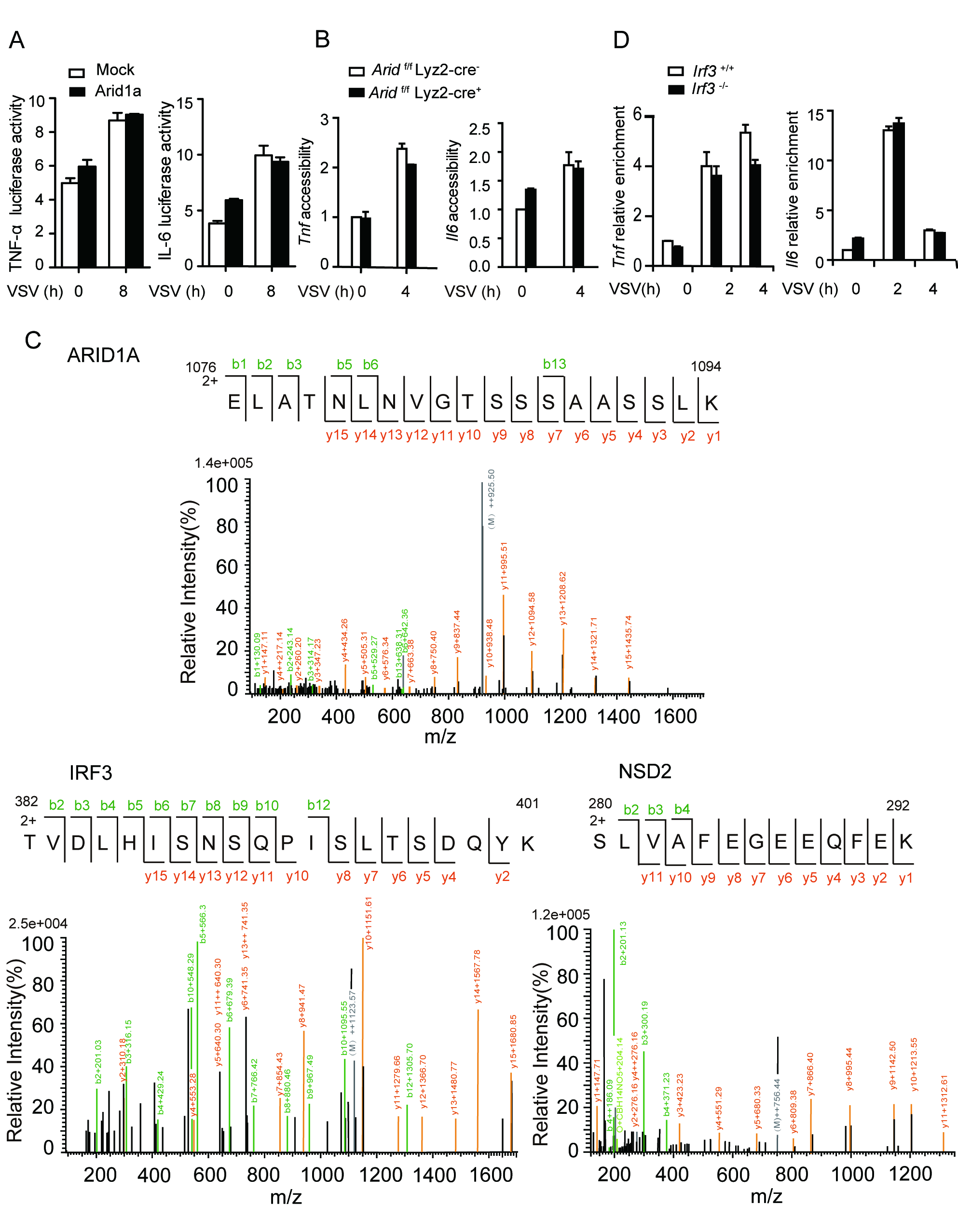

Supplement: Supplementary file 7 — Supplementary Material [file 41419_2021_4032_MOESM7_ESM.tif]

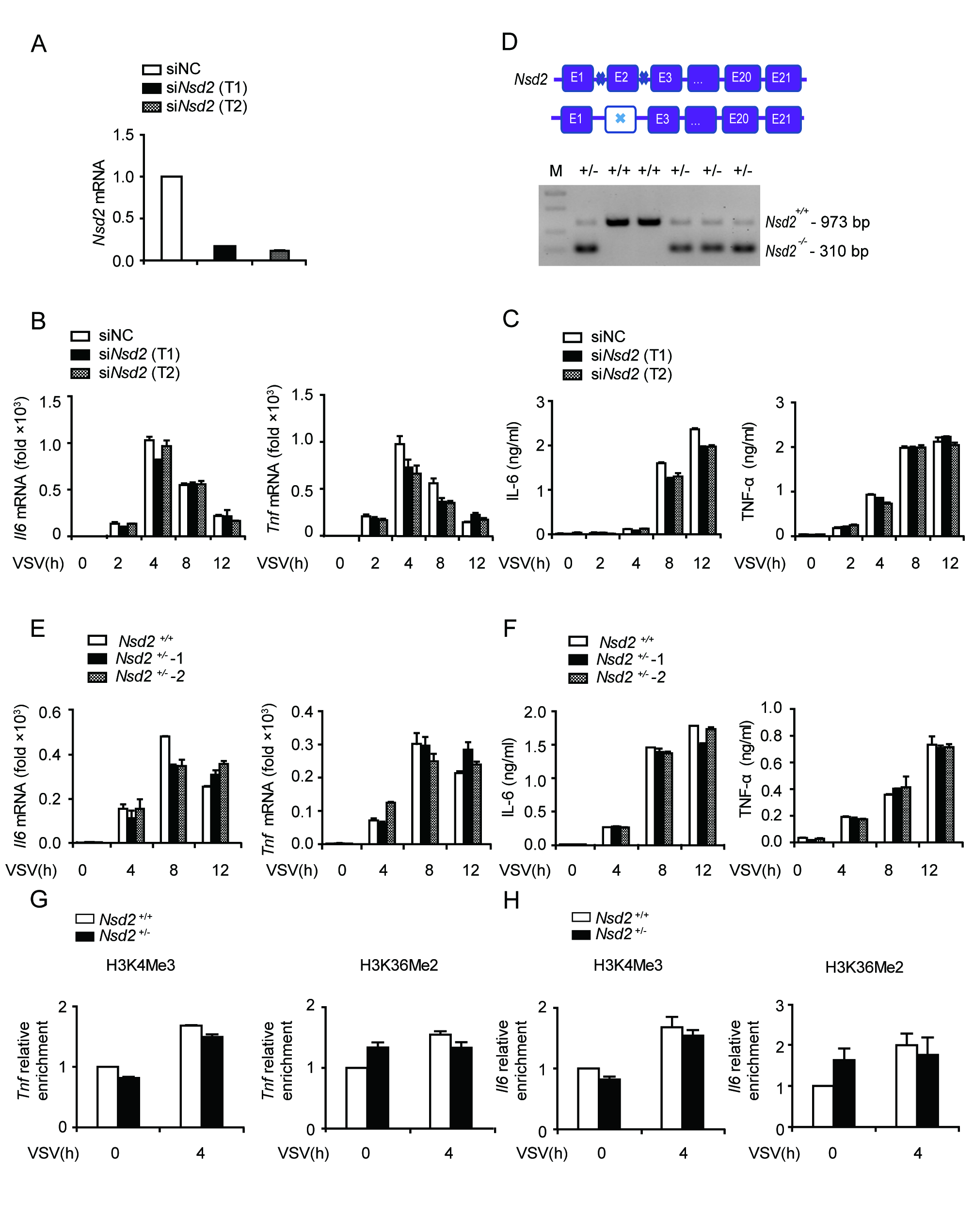

Supplement: Supplementary file 8 — Supplementary Material [file 41419_2021_4032_MOESM8_ESM.tif]

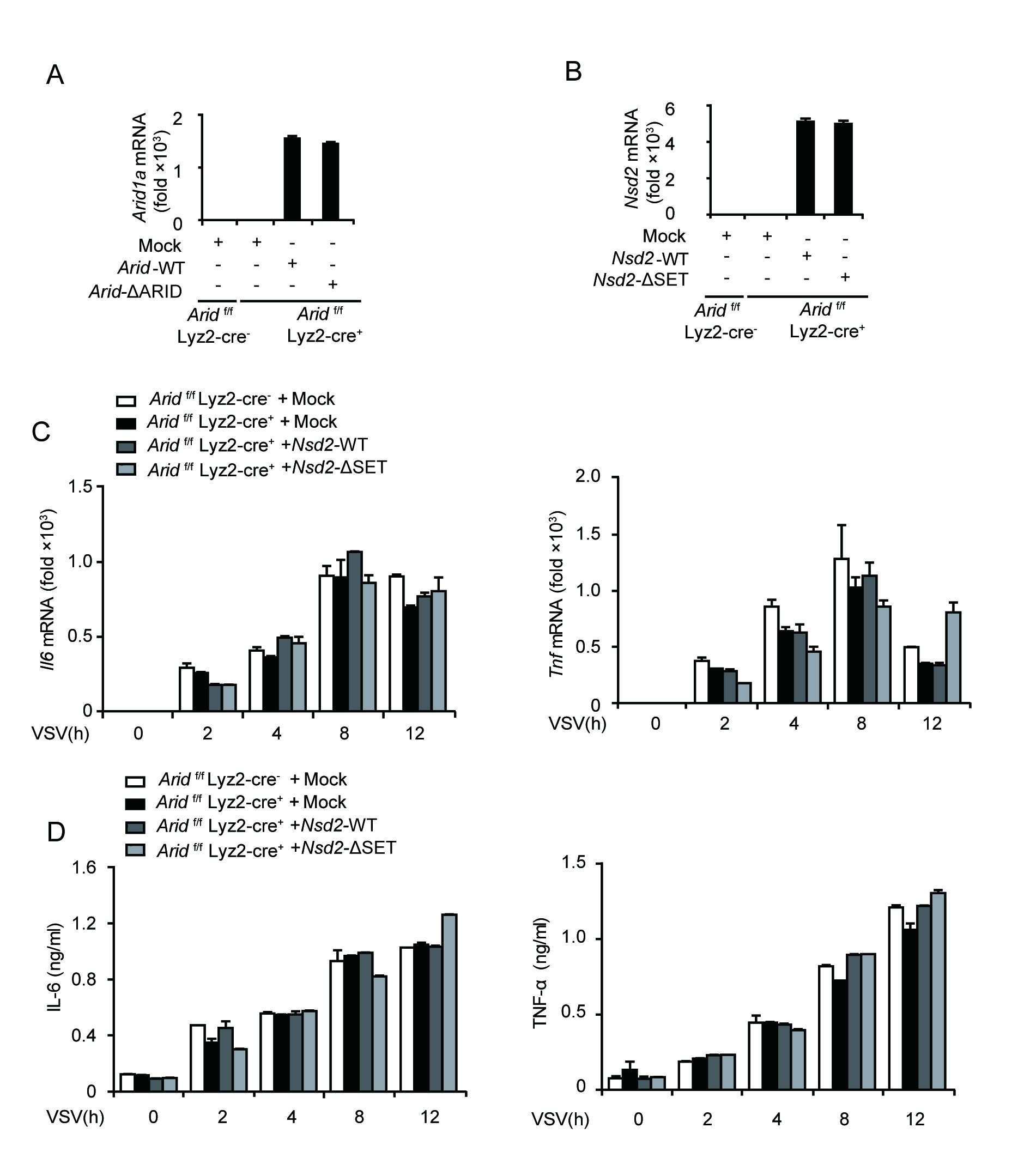

Supplement: Supplementary file 9 — Supplementary Material [file 41419_2021_4032_MOESM9_ESM.tif]
